# DNA and RNA Molecules as a Foundation of Therapy Strategies for Treatment of Cardiovascular Diseases

**DOI:** 10.3390/pharmaceutics15082141

**Published:** 2023-08-15

**Authors:** Ljiljana Rakicevic

**Affiliations:** Institute of Molecular Genetics and Genetic Engineering, University of Belgrade, Vojvode Stepe 444a, 11042 Belgrade, Serbia; ljiljanarakicevic011@gmail.com or lili@imgge.ac.bg.rs; Tel.: +381-062-551-485; Fax: +381-11-3975-808

**Keywords:** personalized medicine, cardiovascular diseases, DNA markers, non-coding RNA, biomarkers

## Abstract

There has always been a tendency of medicine to take an individualised approach to treating patients, but the most significant advances were achieved through the methods of molecular biology, where the nucleic acids are in the limelight. Decades of research of molecular biology resulted in setting medicine on a completely new platform. The most significant current research is related to the possibilities that DNA and RNA analyses can offer in terms of more precise diagnostics and more subtle stratification of patients in order to identify patients for specific therapy treatments. Additionally, principles of structure and functioning of nucleic acids have become a motive for creating entirely new therapy strategies and an innovative generation of drugs. All this also applies to cardiovascular diseases (CVDs) which are the leading cause of mortality in developed countries. This review considers the most up-to-date achievements related to the use of translatory potential of DNA and RNA in treatment of cardiovascular diseases, and considers the challenges and prospects in this field. The foundations which allow the use of translatory potential are also presented. The first part of this review focuses on the potential of the DNA variants which impact conventional therapies and on the DNA variants which are starting points for designing new pharmacotherapeutics. The second part of this review considers the translatory potential of non-coding RNA molecules which can be used to formulate new generations of therapeutics for CVDs.

## 1. Introduction

There has always been a tendency of medicine to take an individualised approach to treating patients (adjusting drug doses according to patient’s weight, selecting antibiotics according to antibiograms, etc.). However, the most significant advances in the field of personalised medicine are related to the development of methods of molecular biology. Methodological approaches of molecular biology have facilitated new and more precise insight into fundamental biological processes, the description of new diagnostic and prognostic markers, recognising new pharmacological targets, as well as setting a base for development of innovative treatment methods. DNA and RNA analyses have enabled more precise diagnostics and more subtle stratification of patients in order to identify patients for specific therapy treatments. All this leads us closer to the ideal of personalised medicine [1]. These research influence therapy strategies for certain diseases in a few ways. Firstly, we are provided with possibilities for more precise diagnostics, more precise prognostics and more precise stratification of patients which allow more successful therapeutic treatment [2,3]. Additionally, the founding and development of pharmacogenetics, which correlates genetics and the effects of certain drugs, have enabled us to reach explanations of interindividual differences in response to given therapy. This creates a basis for better prediction of reaction to certain drugs and more reliable algorithms for treatments [4]. Secondly, advanced research has allowed for the recognition of new pharmacological targets, as well as the formulation of innovative drugs based on the physical and chemical features and function of nucleic acids. What these new formulations promise will affect the essential biological processes as well as overcome the limitations of the currently existing generations of drugs [5]. Whether the protocols for use in already existing pharmacotherapeutics are being improved or entirely new innovative generations of drugs are being created, analyses of DNA and RNA force modern medicine to face both new possibilities and new challenges.

This review considers the most up-to-date achievements related to the use of translatory potential of DNA and RNA in treatment of cardiovascular diseases, and it considers the challenges and prospects in this field. The foundations that enable this translatory potential are also presented. The first part of this review focuses on the potential of the DNA variants which impact conventional therapies and on the DNA variants which are starting points for designing new pharmacotherapeutics. The second part of this review considers the translatory potential of non-coding RNA molecules which can be used to formulate new generations of therapeutics for CVDs.

## 2. Molecular Biology as a Promoter of Research and Treatment of Cardiovascular Diseases

Cardiovascular diseases (CVDs) are a class of illnesses which are a result of concurrence of genetic and acquired factors. They are the leading cause of mortality in developed countries and represent an important medical and social issue, considering their severity and frequency [6,7].

This class of diseases has various manifestations such as hypertension, arrhythmia, venous and arterial thrombosis, heart valve diseases, and myocardial and pericardial diseases etc, which can often be comorbid. Additionally, the presence of other diseases, such as metabolic disorder, hormonal disbalance, rheumatism, infectious diseases, etc., makes diagnostics and treatment even more demanding.

Issues regarding CVDs have spurred research into many directions, the results of which should be a part of the integral platform for treatment and prevention. The attempts of modern society to decrease the frequency of disease and mortality are made through fundamental research, the use of innovative technologies, and education of the population [6,7]. Just as molecular biology has sparked progress over many areas, it set cardiovascular research on an entirely new platform.

### 2.1. DNA Variants—Biomarkers in CVDs

The development of technologies, which has enabled the analysis and interpretation of genetic information, has led to new diagnostic and prognostic possibilities in medicine. The science has been able to describe the causes of monogenic heart diseases [8], as well as the risk factors for diseases such as hypertension [9,10], arrhythmia [11], atherosclerosis [12], and vein thrombosis [13].

Research in the field of pharmacogenetics is particularly important for the development of personalised drug therapy. It is through them that the genes which affect pharmacotherapeutics for cardiovascular system (CVS) have been identified. These genes control the synthesis of enzymes which are necessary for absorption, transport, and metabolism of drugs, as well as proteins which are the targets of pharmacologic substances [4]. In this context, the drugs used against venous and arterial thrombosis-coumarin derivatives and clopidogrel have been the most frequently considered. Both coumarins and clopidogrel are among the most commonly prescribed drugs in today’s medicine. It is known that a division of the patients who take warfarin or clopidogrel can show hypersensitivity or resistance to these drugs, which puts them in life-threatening situations. The anticoagulation drug warfarin, the most prescribed among coumarins, is often referred to as the “poster child” of pharmacogenomics [14,15]. Although there is a large number of genes with an influence on the therapeutic effects of warfarin, it has been shown that of the genes encoding enzymes important for the pharmacodynamics and pharmacokinetics of this drug, these are the ones which are of greatest significance: VKORC1 (the vitamin K epoxide reductase complex subunit 1, pharmacological target of coumarin), P450 2C9 (the major metabolizing enzyme for *S*-warfarin) and P450 4F2 (acts in counterpart to VKORC1 and limits the accumulation of vitamin K). The variants of these genes with the greatest pharmacogenetic potential are the VKORC1*2 (c.-1639G>A; rs9923231), the CYP2C9*2 (c.430C>T; rs1799853), the CYP2C9*3 (c.1075A>C; rs1057910), and the CYP4F2*3 (c.1297G>A; *rs2108622*) [14]. In 2010, the American Food and Drug Administration (FDA) recommended pharmacogenetic testing in relation to warfarin. A recommendation for testing is also given by the Clinical Pharmacogenetics Implementation Consortium (CPIC) [16].

Clopidogrel (along with aspirin) is a cornerstone of antiplatelet therapy and requires enzymatic transformation into an active form of the drug. A number of cytochrome P450 enzymes is involved in the metabolism and activation of clopidogrel. Enzymes CYP1A2, CYP2B6, CYP3A4/5, CYP2C9, and CYP2C19, are involved in the activation, with CYP2C19 being the most engaged. It has been shown that the *CYP2C19* gene variants have the greatest pharmacogenetic potential, namely CYP2C19*2 (681G>A; rs4244285), CYP2C19*3 (636G>A; rs4986893), and CYP2C19*17 (−806C>T; rs12248560) [17,18]. Recommendations for genotype-guided antiplatelet therapy are provided by the CPIC, as well as by the FDA and the DPWG (Dutch Pharmacogenetics Working Group) [19]. The subjects of pharmacogenetic studies were also other drugs relevant to cardiovascular diseases: acenocoumarol, ꞵ blockers, statins, hydralazine, flecainide, and propafenone [20], (listed in Table 1).

Describing DNA markers has an impact on the effects and outcomes of therapy and allow and improvement of the existing therapy protocols. It is possible to design algorithms to predict the effects of a certain therapeutic by analysing pharmacogenetic markers, as well as non-genetic factors which may influence therapy. This enables us to select the safest therapy from drugs produced by the pharmaceutical industry. It has been shown that algorithms designed in this way allow for better predictions than previous interpretations of pharmacogenetic markers given in the form of the FDA-approved warfarin label table [21]. Also, it has been demonstrated that the use of algorithms based on pharmacogenetic analyses is more cost-effective for health systems [22]. Several consortia and working groups have recommended many gene–drug pairs in order to support medical professionals to tailor a unique therapy for an individual patient [16,19,20]. Nevertheless, despite the recommendations and the large amount of generated data, routine pharmacogenetic testing has not yet taken root in practice. Furthermore, it seems that response from the cardiology community is *lukewarm* [23]. Surprisingly, this is true even for clopidogrel which is one of the drugs highly recommended for pharmacogenetic testing. Routine acceptance of recommendations does meet obstacles, mainly due to lack of more precise data on patient adherence, variability in effect of therapy even within the same genotype, and the lack of data on the less frequent groups of patients [23]. The introduction of pharmacogenetic protocols into therapy is often challenged by economic strains, although financial investments which would offer transparent results are not the only issue to be addressed. Meeting these challenges demands innovation in pharmacogenetics and medicine, but also in other areas such as infrastructure, human resources, management, etc [24,25]. This was clearly shown in a study which analysed an attempt to implement pharmacogenetic testing in a large number of medical centres. The study concluded that identified issues were actually non-pharmacogenetic [26].

New challenges have emerged in the field of pharmacogenetics thanks to next-generation sequencing (NGS) [27,28,29]. This technology has enabled for the rapid acceleration in determining the sequence of DNA or RNA, including whole-genome sequencing (WGS), whole-exome sequencing (WES), and transcriptome analyses (RNA-Seq). Chronologically speaking, the first wave of significant results in pharmacogenetics was triggered by studies which focused on genes which were the logical candidates based on their roles in pharmacokinetics and pharmacodynamics of drugs. The studies explained the great deviation of effects that the explored drugs had. Later, with use of technology which allows WES and WGS, the abundance of DNA variants was detected and a large number of them are yet to be defined. The frequency of these variants is smaller [30], but it is expected that once they are interpreted, the distribution of drug responses could be presented as a continuum, rather than a polymodal distribution which corresponds to the pre-NGS era [4].

It is unquestionable that gathering data related to pharmacogenetic markers is a work in progress. The influx of a large amount of data requires tools for their faster analysis, as well as a larger number of studies that can validate potential genetic markers. Also, the integration of data from different fields is of particular importance, and in this sense, the development of bioinformatics and network medicine plays an important role [31].

#### DNA Variants and New Drugs Development

The results of research in the fields of genetics and genomics are also important for the development of new pharmacotherapeutics. It has been shown that proteins whose function is altered as a result of mutations in corresponding genes can be taken into consideration as models or targets for creating new pharmacotherapeutics. These strategies are also taken into consideration for the development of new drugs for several disorders which are closely related to the occurrence of cardiovascular diseases. Often, the focus of such research is on the genetic causes of lipid disorders, which is understandable considering the role lipids have in the development of CVDs [32]. For instance, the gene for PCSK9 (proprotein convertase subtilisin kexin 9), whose specific variants cause familial hypercholesterolemia (FH), as well as its variants lead to striking decreases in low-density lipoprotein cholesterol (LDL-C) and to atherosclerosis risk [33]. In the pharmacological battle against high level of LDL-C, variants of the NPC1L1 (Niemann–Pick C1-like 1) gene are being reconsidered as they have a deactivating effect on proteins, resulting in reduced plasma LDL-C levels and a reduced risk of coronary heart disease [34]. In terms of hyperlipidaemia, the gene of interest is ANGPTL4 (Angiopoietin Like 4) [35,36], while the variations of APOC3 (Apolipoprotein C3) are important for hypertriglyceridemia [37]. The variants of SLC30A8 (Solute Carrier Family 30 Member 8) are significant for the prevention of type 2 diabetes [38].

In general, the influence genomics has on the modern pharmaceutical industry is very strong. Genetic research is increasingly necessary when selecting pharmacological targets and in selecting indications for the process of development of specific pharmacotherapeutic. Certain studies indicate that the success of developing a drug is twofold higher if genetic research has been incorporated into its development [39,40,41].

### 2.2. Non-Coding RNAs (ncRNAs)

**Figure 1:**
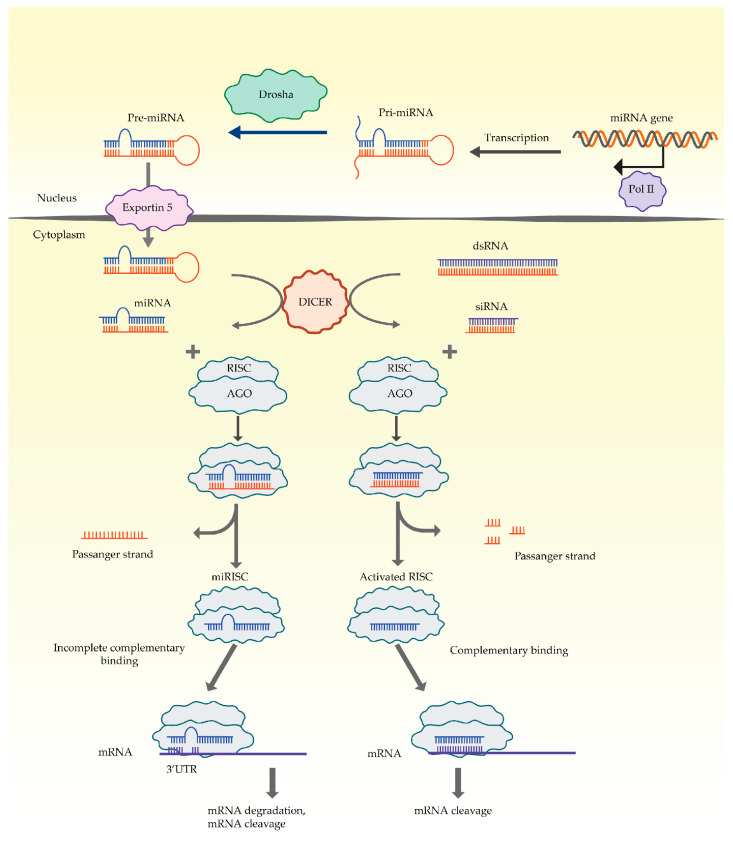
Mechanism of RNA interference. RNAi mechanism recognizes long dsRNA which is then cleaved by Dicer endonuclease into 21–25 nucleotide-long double-strand siRNAs. AGO2 (Argonaute 2) and RISC complexes recognize the new siRNA, it is followed by degradation of sense strand and complementary binding of antisense strand with targeted mRNA which leads to disintegration of mRNA. siRNAs are highly specific with only one mRNA target. MiRNAs biogenesis begins with the synthesis of long primary miRNAs (pri-miRNAs), which are processed into pre-miRNAs by nuclear ribonuclease. Pre-miRNAs are exported from the nucleus, followed by the cleavage by Dicer enzyme and then (as mature miRNA) they are joined to AGO2-RISK complex. The miRNAs bind to the target mRNAs through partial complementary base pairing, leading to translational repression, degradation, and cleavage of mRNAs. Endogenous miRNAs are able to target multiple mRNAs at once.

Messenger RNAs (mRNA), which code sequence of proteins, constitute less than 2% of the expressed genome. The majority of RNAs which are synthesised in the process of transcription are non-coding. Apart from transfer RNA (tRNA) and ribosomal RNA (rRNA) (which were defined first), a large number of non-coding RNAs have been described: long non-coding RNA (lncRNA), micro RNA (miRNA), short interfering RNA (siRNA), PIWI-interacting (piRNA), short nuclear RNA (snRNA), extracellular RNA (exRNA), and small Cajal body associated RNA (scaRNA) [42]. During the 1990s, extensive research on these molecules started and that is when the first mechanisms of expression regulation by non-coding RNA in *Caenorhabditis elegans* were described [43]. Non-coding RNAs were recognised as a special group of molecules essential for maintaining basic biological processes. At the same time, their extraordinary translatory potential in medicine was recognised too. First of all, it became apparent that many non-coding RNAs could be new types of biomarkers and new pharmaceutical targets. In addition, ncRNA research opened possibilities to use the structure and function of nucleic acids for formulating innovative therapeutic strategies and designing entirely novel kinds of therapeutics which could overcome the limitations of present drugs (small molecules, antibodies). In that respect, the principles of complementarity, hybridization, and interference are of the utmost importance [5]. One of the most studied processes in RNA interference is the mechanism for post-transcriptional silencing of gene expression in cells of eukaryotes [44]. Double-stranded RNAs (siRNAs and miRNAs), complementary to the target mRNA, take part in this process. Their presence leads to the activation of a specific RISC (RNA-induced silencing complex) enzyme complex, which results in sequence-specific suppression of gene expression (Figure 1).

NcRNAs were very actively studied in connection to cardiovascular diseases. The goal was to explain the molecular processes which lead to pathological conditions and/or to use principles of the way this class of molecules function in order to find new biomarkers and formulate new generation of drugs.

#### 2.2.1. NcRNAs and Monitoring the Therapy

Some of the possibilities this group of molecules offer are potentially as diagnostically useful biomarkers, monitoring and treating CVDs.

It is known that most of miRNAs in human blood derive from thrombocytes and that they represent promising biomarker candidates [45]. There are data on both basic cellular processes involving thrombocyte miRNAs, as well as on potential significance of miRNAs in relation to analysis of thrombocytes’ activity and the effects of anti-aggregation therapy. MiRNAs in circulation have been shown to indicate the status of thrombocytes in real time and that they can be used as biomarkers for the prediction of various aspects of their function, i.e., they can be used for monitoring and adjusting anti-aggregation therapy [46,47]. Additionally, it has been confirmed that miRNAs affect anticoagulation therapy with coumarin derivatives [48], therapy with direct anticoagulation drugs [49], and that they also affect therapy with statins, i.e., statin intolerance [50]. The predictive potential of long non-coding RNAs has also been recognised for the effects of anti-aggregation therapy [51]. Some databases that provide information regarding associations of ncRNA and drug effects already exist [52]. However, data related to ncRNAs and their association between drug effects are rapidly generated. The traditional biological experiments can recognize new ncRNA–drug-effects associations, but this approach faces challenges like time consumption and financial issues. Additionally, new efforts have been made on predicting the associations between ncRNAs and drugs’ effects by computer methods which offer acceleration of this process and provide new possibilities for improving therapeutic treatments. So, in the near future, we can expect more precise and complete data on this matter [53,54,55].

#### 2.2.2. NcRNAs and New Generation of Therapeutics

RNA therapy is a strategy that involves use of RNA-based molecules for the treatment of diseases. This strategy is based on the use of structural and functional characteristics of RNA molecules, i.e., by mimicking or attenuating their function in the regulation of biological processes. Several types of technologies are used for these approaches: antisense oligonucleotides (ASOs), aptamers, siRNAs, miRNA mimics/attenuation (Table 2).

One of the biggest challenges related to the development of RNA therapy is achieving certain pharmacodynamic and pharmacokinetic properties of drugs. It is achieved by chemical modifications of nucleic acid molecules. The most often employed modifications are phosphate linkage modifications (phosphorothioate) and 2′ ribose modifications such as 2′-O-methyl (2′-OMe), 2′-fluoro (2′-F), 2′-O-methoxyethyl (2′-MOE), and locked nucleic. Also, a subject of great interest for development of the therapy is delivery platforms, i.e., technologies which allow the targeting of drugs to cell-specific ligands. The most considered technology is the binding of different structures to nucleic acid based agents, such as antibody fragments, an entire antibody, or the FDA approved N-Acetylgalactosamine (GalNAc), which binds to asialoglycoprotein receptor 1 (ASGR1), which is highly expressed in the liver [56,57]. The possibilities of RNA therapy use have been researched in a large number of severe diseases, where conventional treatments are not effective and one of the greatest expectations of this therapy is the possible treatment of molecules that have been “undruggable”. Conventional pharmacotherapy mostly uses small molecules (and antibodies in part) and targets different proteins, such as enzymes, ion channels, nuclear receptors, G-protein coupled receptors, etc. A human organism contains about 20,000 proteins, but just a small number of these molecules can be targeted by small-molecule therapy (“druggable” proteins). It is estimated that only about 15% of human proteins are “druggable”, including those already targeted by pharmacotherapeutics [58]. Using RNA-based therapy allows us to act on the upstream phase, i.e., at the level of mRNA, which can turn an “undruggable” protein “druggable”. Additionally, recognising ncRNAs as pharmacological targets ensures a much higher capacity to act with therapeutics. This is due to the fact that more than 70% of the human genome is determined by ncRNAs, in contrast to just 2% determined by human protein [59]. In addition to the use of therapeutics that contain some type of nucleic acids as principle components, the possibility of acting on ncRNAs through small molecules is being considered [60].

The majority of approved RNA drugs for CVDs, as well as RNA drugs in various stages of clinical development, affect the metabolism of lipids. Such drugs are mipomersen, inclisiran, and volanesorsen (Table 3).

*Mipomersen* is an antisense oligonucleotide drug designed to target the apoB-100 mRNA and cause its degradation, thus inhibiting the synthesis of a particular protein. The use of this drug significantly reduces LDL-C and other lipoprotein levels. However, due to the probability of severe adverse effects, including hepatotoxicity, the use of mipomersen is highly limited. It is approved for the treatment of homozygous for familial hypercholesterolemia-autosomal dominant, a genetic disorder that leads to significantly elevated LDL-C levels and increased risk for cardiovascular diseases [61].

*Inclisiran* is a siRNA-based therapeutic that closely mirrors the function of natural, endogenous miRNA. This drug targets mRNA for PCSK9 which is a serine protease that regulates plasma LDL-C levels. Inclisiran is approved for the treatment of adults with primary (familial and non-familial) hypercholesterolemia or mixed dyslipidemia, and for patients with clinical atherosclerotic cardiovascular disease (ASCVD) who require additional lowering of LDL-C. It is intended for administration alone or in combination with other lipid-lowering drugs in patients who are statin-intolerant or patients for whom a statin therapy is contraindicated. During the conducted clinical trials there was no evidence of kidney, liver, muscle, or platelet toxicity [62,63].

*Volanesorsen* is an ASO designed to prevent the translation of apolipoprotein CIII (APOC3) by targeting APOC3 mRNA. Apolipoprotein CIII is a small protein, with a role in the inhibition of triglyceride metabolism and hepatic clearance of chylomicrons, so its overexpression is associated with the risk of atherosclerosis. A combined analysis of several conducted randomised controlled studies has shown that the administration of volanesorsen in patients with severe hypertriglyceridemia significantly reduces triglycerides, very low-density lipoprotein cholesterol (VLDL-C), Apo-B48, non- high-density lipoprotein cholesterol (HDL-C), and increases HDL-C in comparison to placebo. Additionally, volanesorsn, has shown an acceptable safety profile. Most of the registered adverse effects were mild and they were related to injection site reactions. Volanesorsen is approved by the EMA (European Medicines Agency) to treat familial chylomicronaemia syndrome (FCS), a genetic disorder characterised by high levels of triglycerides in the blood. Since FCF belongs to rare diseases, volanesorsen is designated as an orphan medicine (medicines used for the treatment of rare diseases) [64].

Apart from the formulations that have already been approved for therapy, there is a whole range of candidates for the treatment of cardiovascular diseases (Table 3).

*Olpasiran* is a siRNA molecule that prevents translation of apo(a) protein and consequently precludes assembly of the Lp(a) particles. The mechanism of olpasiran action occurs through targeting and degrading mRNA of the LPA gene that encodes apo(a). In the first phase of clinical trials in the first-in-human study, it was shown that the drug was potent in reducing plasma Lp(a) concentration [65,66]. Additionally, several clinical studies are being conducted to evaluate the efficacy, safety, tolerability and the impact of olpasiran on major cardiovascular events (NCT05489614, NCT05481411, NCT04987320, NCT04270760, NCT05581303).

*Pelacarsen*, another drug whose clinical development is still ongoing, is designed according to the principle of ASO action. It is directed against mRNA of the gene for apolipoprotein (a) which consequently inhibits synthesis of apolipoprotein (a) in the liver. This also leads to reduction in the level of Lp (a), which is recognized as an independent cause of CVDs. Interestingly, the use of pelacarsen also reduces LDL-C, apolipoprotein B (apo B), and oxidised phospholipids on apo B and apo(a) [67]. Future studies, as well as re-evaluating laboratory measurement procedures, could give answers to these phenomena. There are also ongoing clinical studies which are examining the effects and safety of this drug (NCT05900141, NCT05646381, NCT05305664, NCT04023552).

*Vupanorsen* is an antisense oligonucleotide that targets the mRNA for angiopoietin like 3 (ANGPTL3) in the liver and consequently inhibits ANGPTL3 protein synthesis. The published results of the first clinical studies show that administration of vupanorsen significantly reduced levels of serum ANGPTL3 protein and some lipid parameters including triglycerides and non–high-density lipoprotein cholesterol in comparison to placebo. Despite these findings, the Pfizer-led vupanorsen clinical development has been discontinued. The rationale behind this decision is that the evident decrease in lipid parameters was still not sufficiently effective to prevent CVDs [68,69].

An agent named *SLN360* is a siRNA that interferes with the biosynthesis of Lp(a). Inhibition of protein synthesis occurs through the formation of a complex between SLN360 and LPA mRNA, which leads to degradation of the targeted mRNA and inhibition of translation (NCT05537571, NCT04606602). According to the published results, SLN360 significantly reduced the plasma Lp(a) concentration, in dose-dependent manner and has a safety profile at a satisfactory level [70]. Another new siRNA agent, *LY3819469*, is still in the clinical development phase. It is designed to influence the level of Lp(a) in the plasma. The ongoing clinical trial aims to investigate the pharmacokinetics and pharmacodynamics of this agent, as well as its safety and tolerability (NCT04914546).

The synthetic agent *MRG-110* is designed to inhibit miRNA92a (NCT03603431). It was previously determined that miRNA92A in human cells performs inhibition of processes necessary for angiogenesis by silencing expression of proangiogenic factors (such as integrin alpha 5), blocking the build up vascular network, and slowing down migration of endothelial cells and their ability to connect to fibronectin. Preclinical studies on mammals have shown that the synthetic miRNA92a inhibitor increases expression of proangiogenic genes, which are targets for miRNA92a [71]. Also, it has been shown that inhibition of miRNK92a improved vascularization after myocardial infarction and blood circulation after hind limb ischemia, a model of peripheral occlusive disease [72]. In an ischemia/reperfusion model in pigs, anti-miR-92a significantly improved cardiac function and vascularization [73,74]. Additionally, it has been demonstrated that this miRNA can be an appropriate therapeutic target for treating cardiac microvascular dysfunction in diabetes [75]. The first results of testing the agent MRG-110 in humans were published in 2020 by Abplanalp and co-authors. In that study, the molecular effects of MRG-110 administration in healthy volunteers were investigated. It was shown that MRG-110 reduced miR-92a in whole blood, circulating CD31^+^ cells, and in extracellular vesicles. Also, this agent has been shown to derepress miRNA92a target genes [76].

The use of synthetic *CDR132L* which inhibits miRNA132 (NCT04045405) has also been approved for testing in humans. MiRNA132 orchestrates the beginning of pathological remodelling of myocard by silencing a range of relevant genes such as *NOS3* (Endothelial Nitric Oxide Synthase 3) and *SERCA2a* (Sarcoplasmic/Endoplasmic Reticulum Ca^2+^ATPase 2a). During the preclinical studies, it was shown that CDR132L blocks adverse remodelling of myocard and restores coronary function, while, on a molecular level, it restores the expression of ATPase SERCA2a which is crucial for retaking in calcium during contractions of cardiomyocytes [77]. The results of the first application of CDR132L in humans were published in 2021 by Taubel and co-authors. They conducted a Phase 1b randomized, double-blind, placebo-controlled study in patients with heart failure and showed that CDR132L was safe and well tolerated; furthermore, the study showed that the use of CDR132L preparation leads to cardiac functional improvements-improvement in left ventricular ejection fraction (LVEF), significant QRS narrowing, reduction in the amino terminal fragment of pro-brain natriuretic peptide (NTproBNP) levels, and positive trends for relevant cardiac fibrosis biomarkers [78].

#### 2.2.3. NcRNAs and Extracellular Vesicles

A particularly important aspect of the translation potential of non-coding RNAs is their natural presence in extracellular vesicles (EVs). EVs are released by all living cells. They are surrounded by a double membrane and contain numerous molecules, such as proteins, nucleic acids, bioactive lipids, etc. EVs are important mediators in intercellular and inter-organ communication which uses their contents to pass different signals to targeted cells and they participate in different processes within those cells [79]. Based on their biogenesis, size, and physicochemical properties, EVs can be classified into subcategories (exosomes, microvesicles, apoptotic bodies). The International Society of Extracellular Vesicles recommended defining all prepared vesicles (independent of their origin) as EVs [80].

There is a lot of available data related to the role of molecular mediators carried by exosomes originating from the heart as well as data on mediators carried by exosomes from other organs that affect the heart [81,82]. EVs are especially interesting for their potential regarding the regeneration of myocard [83,84]. The research on miRNA and lncRNA which originated from EVs is important because extracellular vesicles affect myocard with these two classes of non-coding RNAs [85].

EV-derived ncRNAs are a part of a wide range of cellular processes and many aspects of their functions may be considered for new therapeutic strategies. It has been shown that myocardial infarction is associated with increased levels of lncRNA, NEAT1, and miR-328-3p [86,87], as well as with decreased levels of lncRNA HCP5, miR-21, miR-24, miR-98-5p, miR-150p, miR-185, and miR-212-5p [88,89,90,91,92,93,94]. Additionally, there is enough convincing evidence related to heart damaged by infarction, which shows that restoring levels of decreased ncRNAs back to normal can be achieved by using ES-derived ncRNAs [88,89,90,91,94,95]. Experiments with models for myocardial infarction have also shown that ncRNAs derived from EVs are involved in processes which are essential for recovery of the heart, i.e., they increase cardiomyocyte survival and cardiac functional recovery [89,92,94,95,96,97,98,99,100,101]. Also, it has been confirmed that EV-derived ncRNAs influence other processes related to the cardiovascular system such as inflammatory response [90,95,102,103,104], as well as vascular protection and angiogenesis [105,106,107,108,109,110,111].

The results of this research indicate the great translatory potential of EVs. As natural delivery systems, EVs act as a safe vehicle because they allow for the stabilization and protection of ncRNAs (as well as other molecules they transfer) on the way to targeted cells. This, and their biocompatibility and targeting ability, makes them very attractive for innovative therapy technologies. On the other hand, there are a number of obstacles related to the biological production methods of EVs in large-scale production. The greatest challenges are related to isolation of undamaged EVs, purity and characterization, and high costs. However, a large number of ongoing studies promise to solve the mentioned problems in the near future [112].

The number of data related to non-coding RNAs is rapidly growing and it is obvious that various types of non-coding RNAs are joined into unique regulatory processes. This makes both setting the premises of research and the interpretation of results very complex. For that very reason, bioinformatic research is so important. It can facilitate extractions of clusters of molecules which are most important to the study of specific physiological and pathophysiological processes. At the same time, it would facilitate the selection of highly specific molecules related to specific diseases as the most relevant biomarkers or pharmacological targets [113].

## 3. Conclusions and Future Perspectives

It is usual to simultaneously use various kinds of therapies to target various pathogenic pathways when tackling problems which develop with CVDs. At the same time, it is still necessary to develop new treatments which can give better treatment results, and modern research into DNA and RNA molecules, and their role in new therapeutic approaches, makes a great contribution to this. The development of pharmacogenetics has led to the identification of DNA markers that allow more precise classification of patients to sub-phenotypes, which is important for more successful application of conventional therapy. Constant advancement in this field and the generation of new data forges a basis for designing multi-variant panels which can be used for prediction of a wide range of drug-induced adverse effects. We can also expect panels encompassing RNA pharmacogenetic markers. Additionally, studying the principals of RNA function led to formulations of innovative therapeutic strategies such as RNA therapies. Probably the most significant achievement of RNA based therapy so far is the possibility to influence molecules which have been “undruggable” until now. This field is rapidly advancing and it is followed by a huge data generation in fundamental and preclinical research. Investments in multi-omics research, as well as in non-biological disciplines such as computer science, should enable us to faster identify the most adequate molecule candidates for the further development of drugs.

Generally, modern medicine confronts a large quantity of data related to translatory potential of DNA and RNA molecules for treatment of cardiovascular diseases. However, new challenges have emerged simultaneously with it. For instance, the speed of generating data and new findings is not in proportion to their practical application. In a way, more subtle stratification on a molecular level makes cardiovascular diseases, which are already of complex nature, even more complex for consideration. A deluge of information related to DNA and RNA poses questions about the management and interpretation of results, about recognising specificities of potential biomarkers, and about the possibilities of formulating drugs which are safe for human use. Also, there are questions related to infrastructural and economic components of analysing a large number of potential biomarkers which may become an important element of comprehensive strategies for the diagnostics, monitoring, and treatment of diseases.

**Figure 2:**
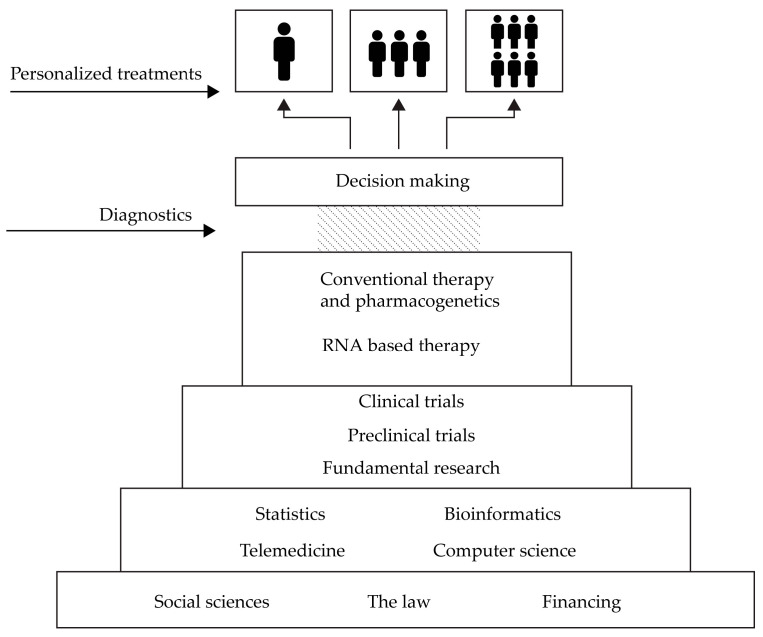
Different areas and activities involved in the development of personalized therapeutic approaches.

Overcoming these challenges demands social investments and the engagement of the scientific community on various levels (Figure 2). First of all, data processing and interpretation require employing within computer science and bioinformatics at proportional level. Also, it is necessary to continue studies in order to research and validate DNA and RNA biomarkers with high translatory potential in supervising treatment, as well as to research possible therapeutics for CDV. The cardiology community needs additional studies even for well defined DNA markers in pharmacogenetics in order to provide missing data (mentioned above)—precise medicine demands precise data and precise conclusions. At the same time, we are looking for efficient modes for the use of well-defined pharmacogenetic markers in routine practice. Finding these modes is a challenge even for developed economies and demands innovation in medicine and genetics, as well as innovation in the entire management of health systems.

However, despite challenges, trends such as intensive development of bioinformatics tools and artificial intelligence, constant improvement of technological approaches to molecular biology analyses, as well as decreases in costs of analyses offer satisfactory answers. Finally, all present knowledge indicates that the foundation for developing the medicine of the future has already been laid, and DNA and RNA molecules play an important role in it.

## Figures and Tables

**Table 1:** The most relevant gene for pharmacogenetics in CVDs.

Farmacogenetics Factors	Drugs	Adverse Effects (Related to Genetic Factors)	Recommendations for Pharmacogenetics Testing
*VKORC1*, *CYP2C9*, *CYP4F2*	Coumarin derivatives warfarin acenocoumarol	Overdose (bleeding) Resistance (thrombosis)	CPIC*, FDA** DPWG***
*CYP2C19*	Clopidogrel	Overdose (bleeding) Resistance (thrombosis)	CPIC, FDA
*SLCO1B1*	Statins simvastatin atorvastatin	Myopathy	CPIC DPWG
*ADRB1*	β-Adrenergic receptor antagonists metoprolol	Bradycardia	DPWG
*CYP2D6*	Antiarrhythmics flecainide, propafenone	Drug accumulation	DPWG DPWG, FDA

CPIC*—Clinical Pharmacogenetics Implementation Consortium; FDA**—Food and Drug Administration; DPWG***—Dutch Pharmacogenetics Working Group.

**Table 2:** Technologies employed in RNA therapy development.

Formulatrion	Fundamental Basic
ASO	ASO are short, synthetic, single-stranded oligonucleotides that can be based on both DNA and RNA. ASOs bind their target RNAs in a sequence-specific manner modulating mRNA function and gene expression or inactivating miRNAs.
Aptamers	Aptamers are short, artificial single-stranded oligonucleotides, composed of DNA or RNA, that bind target molecules (proteins, peptides, carbohydrates, small molecules). The important quality of these molecules is conformation, which allows high affinity and high specificity towards ligands. That is precisely why aptamers are often referred to as chemical antibodies (aptemer derives from Latin “aptus” meaning “to fit”).
siRNAs	SiRNAs are non-coding RNAs which are involved in RNA interference (RNAi). Synthetic siRNAs are 20–25 base pairs with 3′ overhangs long and they can avoid the action of Dicer enzyme directly engaging in RISC and control of gene expression.
MiRNA mimics/atenuation	MiRNAs are non-coding RNA involved in RNA interference. Endogenous miRNAs are able to target multiple mRNAs at once. The miRNA mimics is designed to have the same sequence as the endogenous miRNA and can target multiple mRNAs at once. Anti-miRNAs are ASOs designed to be (fully or partially) complementary to a selected endogenous miRNA to prevent interaction with its target genes.

**Table 3:** RNA-based formulations for treatment of CVSs in humans.

Phase	Drug (Brend Name)	Chemical Specificity and Modification	Target
Approved for therapy (by FDA or/and EMA)	Mipomersen Kynamro^®^	ASO PS; 2′-MOE	*APOB* mRNA
	Inclisiran Leqvio^®^	siRNA 2′-F; 2′-MOE; 2′-O-Me; PS; GalNAc	*PCSK9* mRNA
	Volanesorsen Waylivra^®^	ASO 2′-MOE	*APOC3* mRNA
Various stages of clinical development	Olpasiran	siRNA PS; 2′-O-Me; 2′-F; GalNAc	*LPA* mRNA
	Pelacarsen	ASO 2′-O-MOE; GalNAc	*LPA* mRNA
	Vupanorsen	ASO GalNAc	*ANGPTL3* mRNA
	SLN360	siRNA 2′-O-Me; 2′-deoxy-2′-F; GalNAc	*LPA* mRNA;
	LY3819469	siRNA 2′-O-Me; 2′-F; GalNAc	*LPA* mRNA;
	MRG-110	Anti-mir	miRNA-92A
	CDR132L	Anti-mir	miRNA-132

2′-O-Me, 2′-methoxy; 2′-O-MOE, 2′-methoxyethyl; 2′-F, 2′-fluoro; PS, phosphorothioate; GalNAc, N–Acetylgalactosamine.

## Data Availability

Not applicable.
